# Unassembled cell wall proteins form aggregates in the extracellular space of *Chlamydomonas reinhardtii* strain UVM4

**DOI:** 10.1007/s00253-022-11960-9

**Published:** 2022-05-23

**Authors:** Lorenzo Barolo, Audrey S. Commault, Raffaela M. Abbriano, Matthew P. Padula, Mikael Kim, Unnikrishnan Kuzhiumparambil, Peter J. Ralph, Mathieu Pernice

**Affiliations:** 1 Climate Change Cluster, University of Technology Sydney, 15 Broadway, Ultimo, Sydney, NSW 2007 Australia; 2 School of Life Sciences and Proteomics Core Facility, Faculty of Science, University of Technology Sydney, 15 Broadway, Ultimo, Sydney, NSW 2007 Australia

**Keywords:** Microalgae, Recombinant protein, Secretome, *Chlamydomonas*, Glycoproteins, Proteomics, Pherophorins

## Abstract

**Abstract:**

The green microalga *Chlamydomonas reinhardtii* is emerging as a promising cell biofactory for secreted recombinant protein (RP) production. In recent years, the generation of the broadly used cell wall–deficient mutant strain UVM4 has allowed for a drastic increase in secreted RP yields. However, purification of secreted RPs from the extracellular space of *C. reinhardtii* strain UVM4 is challenging. Previous studies suggest that secreted RPs are trapped in a matrix of cell wall protein aggregates populating the secretome of strain UVM4, making it difficult to isolate and purify the RPs. To better understand the nature and behaviour of these extracellular protein aggregates, we analysed and compared the extracellular proteome of the strain UVM4 to its cell-walled ancestor, *C. reinhardtii* strain 137c. When grown under the same conditions, strain UVM4 produced a unique extracellular proteomic profile, including a higher abundance of secreted cell wall glycoproteins. Further characterization of high molecular weight extracellular protein aggregates in strain UVM4 revealed that they are largely comprised of pherophorins, a specific class of cell wall glycoproteins. Our results offer important new insights into the extracellular space of strain UVM4, including strain-specific secreted cell wall proteins and the composition of the aggregates possibly related to impaired RP purification. The discovery of pherophorins as a major component of extracellular protein aggregates will inform future strategies to remove or prevent aggregate formation, enhance purification of secreted RPs, and improve yields of recombinant biopharmaceuticals in this emerging cell biofactory.

**Key points:**

• *Extracellular protein aggregates hinder purification of recombinant proteins in C. reinhardtii*

• *Unassembled cell wall pherophorins are major components of extracellular protein aggregates*

• *Known aggregate composition informs future strategies for recombinant protein purification*

**Supplementary Information:**

The online version contains supplementary material available at 10.1007/s00253-022-11960-9.

## Introduction

Over the past 20 years, the chlorophyte *Chlamydomonas reinhardtii* has emerged as a promising alternative biofactory to produce recombinant proteins (RPs) (Eichler-Stahlberg et al. 2009; Rasala and Mayfield 2011; Barolo et al. 2020). *C. reinhardtii* possesses economically interesting features, such as high growth rates and minimal culture media requirements (Mathieu-Rivet et al. 2014). Unlike mammalian cells, *C. reinhardtii* is not susceptible to contamination by human pathogens and is generally recognized as safe (GRAS) (Specht et al. 2010). *C. reinhardtii* also shares a similar post-translational machinery with human cells, facilitating production of complex RPs that require post-translational processing and modification to enhance their stability and functional activity (De Muynck et al. 2010; Mathieu-Rivet et al. 2014; Fahad et al. 2015; Rasala and Mayfield 2015). These advantages, combined with the availability of a fully sequenced genome and an advanced genetic toolkit (Specht et al. 2010), make *C. reinhardtii* the most comprehensive microalgal platform for expression of RPs.

RP production in *C. reinhardtii* can be achieved in both the chloroplast and the nucleus. While the level of chloroplastic transgene expression can reach 20% of total soluble protein (Mathieu-Rivet et al. 2014), the chloroplast lacks the required machinery to perform post-translational modifications (PTMs) such as glycosylation (Specht et al. 2010; Fahad et al. 2015). Nuclear-encoded proteins, on the other hand, can be glycosylated through the secretory pathway to reach their final stable and active form (Baier et al. 2018). Glycosylation directly and indirectly influences yield, efficacy, and pharmacokinetics of RPs (Barolo et al. 2020; Mizukami et al. 2018), making nuclear transformation the method of choice to produce active complex recombinant biopharmaceuticals such as antibodies (Solá and Griebenow 2009). However, low nuclear transgene expression in *C. reinhardtii* still represents a major issue for industrial RP production (Neupert et al. 2009). Many different strategies have been attempted over time to achieve efficient nuclear expression in *C. reinhardtii*, from the development of new promoters to the addition of functional peptides to improve expression and allow secretion (Schroda et al. 2000; Rasala et al. 2012; Molino et al. 2018). A major advance was the generation by Neupert et al. (2009) of the UVM4 strain, a cell wall–deficient UV-mutated *C. reinhardtii* cell line with improved RP production. The authors selected the arginine-auxotrophic cell wall–deficient *C. reinhardtii* strain CC-4350 (also known as cwd *mt* + arg7), and subjected it to genetic engineering to restore arginine prototrophy and to allow transgene expression measurement. A positive strain (cell wall–deficient arginine-prototrophic strain showing overexpression of the inserted gene), known as Elow47, was then subjected to UV mutagenesis to obtain a mutated cell line with improved RP production. Two of the mutant strains (UMV4 and UVM11) generated were found to have increased expression of intracellular recombinant GFP and YFP, reaching 0.2% of total soluble proteins (Neupert et al. 2009). Strain UMV4 has since been widely exploited for intracellular and secreted RP production, achieving significantly improved yields of secreted RP (12–15 mg/L) (Lauersen et al. 2015; Ramos-Martinez et al. 2017). These higher yields of secreted RP, combined with the absence of a cell wall that greatly facilitates genetic transformation of strain UVM4 (Kindle 1990), explains the wide use of this strain for RP production.

Despite significant advances towards more efficient nuclear transgene expression in *C. reinhardtii*, strain UVM4 presents a major disadvantage: the purification of secreted RPs accumulated in the extracellular space is challenging. Secreted RPs produced in strain UVM4 appear to be “trapped” in an extracellular matrix, impeding proper migration on electrophoresis gels, proper binding of antibodies, and successful purification using liquid chromatography methods. Multiple studies associated impaired purification of recombinant proteins from the secretome of strain UVM4 with an extensive amount of cell wall protein aggregates populating the extracellular space of this strain (Zhang and Robinson 1990; Baier et al. 2018; Eichler-Stahlberg et al. 2009). Previous studies have reported that cell wall proteins are still successfully produced in cell wall–deficient strains; however, they do not assemble in the multi-layered cell wall and they are subsequently released into the extracellular space (Davies and Plaskitt 1971; Voigt et al. 1997; Cronmiller et al. 2019). When released and accumulated in the extracellular space, cell wall proteins can form aggregates that might trap the RP and consequently affect its purification (Baier et al. 2018; Zhang and Robinson 1990). Therefore, although cell wall deficiency is beneficial for increased transformation efficiency, it can be linked to impaired RP purification and yields (Baier et al. 2018). Baier et al. circumvented this issue by fusing an amphiphilic hydrophobin protein tag to their secreted RP and using a detergent-based aqueous two-phase protein extraction system as the first step of purification prior to affinity chromatography (Baier et al. 2018). However, the final yield of purified RP was still low (15 µg L^−1^) and the method is difficult to scale-up, highlighting the need for additional strategies to address this purification challenge.

A thorough analysis of the extracellular space of strain UVM4 versus its parental strain would allow for the identification of the proteins possibly involved in the formation of extracellular aggregates. However, we were unable to locate any data on secreted recombinant protein production in the immediate predecessors to strain UVM4 (cell wall–deficient strain CC-4350 and its derivative Elow47), and therefore it is unknown whether the secretomes of these strains also contain protein aggregations that may impair RP purification. Moreover, both strains CC-4350 and Elow47 are cell wall–deficient. A comparison with a cell-walled control would widen the information gathered on the secretome of cell wall–deficient strains such as UVM4 (as done previously for other cell wall–deficient strains (Zhang and Robinson 1990)). Given that the aim of this study is to understand the proteomic landscape that accompanies the cell wall–deficient phenotype and contributes to extracellular aggregate formation, the closest ancestral strain possessing a cell wall (*C. reinhardtii* wild type strain 137c) was chosen as the control to characterise strain UVM4 secretome and to identify the possible causes for impaired RP purification in this strain.

Here, we conduct the first analysis of the secretome of the cell wall–deficient UV-mutated *C. reinhardtii* strain UVM4, compared to its cell-walled ancestor strain 137c, to identify the dominant extracellular proteins that may be involved in hindering RP purification. Both strains are widely used today for RP production in *C. reinhardtii*. The information gathered in this study will help inform genetic approaches such as knock-out/knock-down of cell wall proteins to facilitate the purification of RPs in the biotechnologically relevant and widespread *C. reinhardtii* strain UVM4.

## Materials and methods

### C. reinhardtii cultivation conditions and harvesting

*C. reinhardtii* wild type strain 137c (CC-125 *mt* +) was purchased from Invitrogen, Waltham, Massachusetts, USA (GeneArt® Chlamydomonas Protein Expression Kit) (*C. reinhardtii* strain CC-125 *mt* + can be obtained online at the Chlamydomonas Resource Center, St. Paul, Massachusetts, USA, https://www.chlamycollection.org/). *C. reinhardtii* strain UVM4 was graciously provided by Prof. Ralph Bock. Historically, strain UVM4 was obtained after genetic engineering and UV mutagenesis of strain CC-4350, which was isolated by Fernández and Matagne (1984; 1986) from a cross between a cell wall–deficient strain obtained from Davies and Plaskitt (1971) (from the cw15 family) and an *arg7-8 nit-1 nit-2* strain from the laboratory of Prof. Matagne. Both strain cw15 and *arg7-8 nit-1 nit-2* were derived from strain CC-125 *mt* + (also named strain 137c) (Davies and Plaskitt 1971; Pröschold et al. 2005) (Prof. Matagne dir. comm.). *C. reinhardtii* 137c and UVM4 strains were up-scaled from a single colony and grown under mixotrophic conditions at 25 °C and 100 rpm in 4 mL Tris acetate phosphate (TAP) medium (Gorman and Levine 1965) with ~ 50 µmol photons/m^2^/s^1^ of continuous light until mid-exponential phase (OD_750 nm_ ≈ 1.5 Absorbance Units (AU)). The cells were then transferred (1:250 inoculum ratio) to 500 mL of TAP medium (in a total of 6 different flasks, 3 flasks for strain 137c and 3 flasks for strain UVM4) and grown under the same conditions. Samples (cells and medium) were harvested after 72 h (OD_750 nm_ ≈ 1.5 AU). *C. reinhardtii* cells and medium were separated by tangential flow microfiltration with a 0.2 µm polyethersulfone (PES) membrane (Vivaflow 200, Sartorius, Göttingen, Germany) at the lowest speed. The filtrate (growth medium) was subsequently concentrated 250-fold by crossflow ultrafiltration with a 3 kDa cutoff (3000 MWCO PES membrane, Vivaflow 200 and Vivaspin Turbo 15, Sartorius, Göttingen, Germany). Samples were stored at − 80 °C. The experiment was performed with three independent biological replicates for each strain. To validate the harvesting method and to prove that the tangential flow microfiltration was not disrupting living cells (consequently altering the intracellular and extracellular proteomes), the ratio of dead to live cells was monitored before and after the filtration, using the LIVE/DEAD® Fixable Violet Dead Cell Stain (ThermoFisher Scientific, Waltham, Massachusetts, USA) and analysed by flow cytometry (Supplementary Fig. S1).

### Cell morphology and motility

To unveil the differences in cell morphology between strain 137c and strain UVM4, the cells of both strains were analysed by light and electron microscopy. Strain 137c and strain UVM4 cells were harvested after 72 h (mid-exponential phase) by centrifugation at 1000 × g for 5 min and fixed with 1% formaldehyde and 2.5% glutaraldehyde in phosphate buffered-saline (PBS: 0.1 M phosphate, 0.6 M sucrose, pH 7.5) for 24 h at 4 °C. Samples were then rinsed and stored in 1 × PBS at 4 °C until further analysis using light microscope, scanning electron microscope (SEM) and transmission electron microscope (TEM). For light microscopy, 10 µL of culture was deposited on microscope slide, covered with a coverslip and then imaged via light microscope (Nikon Eclipse Ci-L, Nikon Instruments Inc, Tokyo, Japan). For SEM, the strains were diluted 100 times in a 50:50 1 × PBS: Milli-Q water mix and 1 µL of culture was deposited onto formvar-coated copper finder grids (Emgrid Australia, Gulfview Heights, South Australia, Australia) and dried for 24 h in a desiccator. Immediately prior to imaging, the grids were mounted onto an aluminium stub, coated in 10 nm carbon (Leica EM ACE600 High Vacuum Coater, Leica microsystems, Wetzlar, Germany), and imaged with an in lens secondary electron detector at 5 kV in a field emission a SEM (Zeiss Supra 55VP SEM, Zeiss, Oberkochen, Germany) at the Microstructural Analysis Unit, UTS. For TEM, samples were transported to the Australian Centre for Microscopy and Microanalysis at the University of Sydney for embedding in SPURR resin. Briefly, cell pellets were post-fixed in 1% OsO_4_ for 1 h, rinsed in Milli-Q water, then dehydrated in an increasing gradient of ethanol (50%, 70%, 90%, 100%) followed by 100% acetone. Infiltration with SPURR resin was achieved via an increasing gradient of SPURR resin (25%, 50%, 75%, 90% and 100%) mixed with acetone, including an overnight infiltration session with fresh 100% SPURR resin. The samples were then placed in a 65 °C oven for 24 h to allow for polymerisation. Ultra-thin sections (~ 100 nm thick) were cut using a diamond knife and placed onto formvar-coated copper finder grids (Emgrid Australia, Australia) and imaged using TEM (Philips CM200, FEI, Hillsboro, Oregon, USA) at the University of New South Wales (UNSW, Australia). Semi-thin Sects. (500 nm thick) were also used for further observations of cell cross sections and size measurement. Briefly, semi-thin Sects. (500 nm thick) were obtained by sectioning resin blocks, using a glass blade on an ultramicrotome (Leica EM UC6, Leica Microsystems, Wetzlar, Germany), and stained using a 5% toluidine blue solution (5% toluidine blue, 10% Na_2_B_4_O_7_, ultrapure water). Image analysis was carried out using the Eclipse Ci-L microscope (Nikon, Tokyo, Japan) and the images were captured and calibrated of scale bar using the Infinity Analyse software. Cell size of both strains was measured using ImageJ Software (*n* = 30).

### Protein extraction and sample preparation

To remove non-protein contaminants, the proteins in the 250-fold concentrated extracellular fractions were precipitated using a chloroform/methanol precipitation protocol adapted from Wessel and Flügge (1984) and subsequently resuspended in 100 mM of triethylammonium bicarbonate buffer (TEAB) with 1 M of urea. The concentration of proteins was quantified with a bicinchoninic (BCA) protein assay (Pierce™ BCA Protein Assay Kit, ThermoFisher Scientific, Waltham, Massachusetts, USA). A portion of the proteins was kept aside to be visualised by gel electrophoresis and treated for glycans analysis; the rest was further process for mass spectrometry analysis as follow. The protein preparation step (reduction and alkylation) was performed using 5 mM of tris(2-carboxyethyl)phosphine (TCEP) and 20 mM of acrylamide monomers (AM), while 20 mM of dithiothreitol (DTT) was used to quench the alkylation reaction. To remove impurities, 40 µg of total protein was subjected to a single-pot, solid-phase-enhanced sample preparation (SP3) as described in Hughes et al. (2019). After SP3 preparation, samples were resuspended in 100 µL of 200 mM ammonium bicarbonate (AMBIC), obtaining a final concentration of protein of 0.4 µg/µL in all samples. Finally, protein digestion was performed overnight with proteomic grade trypsin (Trypsin Gold, Promega, Madison, Wisconsin, USA) in a 1:50 w/w ratio at 37 °C. The peptides obtained from digestion were quantified using Pierce™ Quantitative Colorimetric Peptide Assay (ThermoFisher Scientific, Waltham, Massachusetts, USA). 2 µg of peptides was concentrated and resuspended in 5 µL of mass spectrometry loading buffer (2% acetonitrile (ACN), 0.2% trifluoroacetic acid (TFA)) and analysed by mass spectrometry.

### Mass spectrometry proteome analysis

Using an Acquity M-class nanoLC system (Waters, Milford, Massachusetts, USA), 5 µL of the sample was loaded at 15 µL/min for 2 min onto a nanoEase Symmetry C18 trapping column (180 µm × 20 mm) before being washed onto a PicoFrit column (75 µm ID × 300 mm; New Objective, Woburn, Massachusetts, USA) packed with Magic C18AQ resin (3 µm, Michrom Bioresources, Auburn, California, USA). Peptides were eluted from the column and into the source of a Q Exactive Plus mass spectrometer (Thermo Scientific, Waltham, Massachusetts, USA) using two buffers: MS buffer A (100% H_2_O + 0.1% formic acid) and MS buffer B (100% acetonitrile + 0.1% formic acid) with the following program: 5–30% MS buffer B over 90 min, 30–80% MS buffer B over 3 min, 80% MS buffer B for 2 min, and 80–5% MS buffer B for 3 min. The eluting peptides were ionised at 2400 V. A data-dependant MS/MS (dd-MS2) experiment was performed, with a survey scan of 350–1500 Da performed at 70,000 resolution for peptides of charge state 2 + or higher with an AGC target of 3e^6^ and maximum injection time of 50 ms. The Top 12 peptides were selected fragmented in the Higher-energy C-trap dissociation (HCD) cell using an isolation window of 1.4 m/z, an AGC target of 1e^5^ and maximum injection time of 100 ms. Fragments were scanned in the Q Exactive Plus mass spectrometer (Thermo Scientific, Waltham, Massachusetts, USA) analyser at 17,500 resolution and the product ion fragment masses measured over a mass range of 50–2000 Da. The mass of the precursor peptide was then excluded for 30 s. The mass spectrometry proteomics data have been deposited to the ProteomeXchange Consortium via the PRIDE (Perez-Riverol et al. 2022) partner repository with the dataset identifier PXD032195 and 10.6019/PXD032195. The MS/MS data files were searched using Peaks Studio X (Han et al. 2011) against the UniProt *Chlamydomonas reinhardtii* proteome (UP000006906, protein count: 18,829) and a database of common contaminants with the following parameter settings: Fixed modifications: none; variable modifications: oxidised methionine, deamidated asparagine; enzyme: semi-trypsin; number of allowed missed cleavages: 3; peptide mass tolerance: 10 ppm; MS/MS mass tolerance: 0.05 Da. The results of the search were then filtered to include peptides with a –log_10_(P_value_) score that was determined by the false discovery rate (FDR) of < 1%, the score being that where decoy database search matches were < 1% of the total matches.

### Protein annotation

Extracellular proteins were quantified using PEAKS label-free quantification. Label-free quantification uses peptide features (such as quality of peptide detection, mass-to-charge ratio, and LC retention time) to match peptides from different samples, and uses signal intensities (peak areas) to calculate fold change between samples. These parameters were measured for 3 biological replicates for each strain and subsequently compared between the two different strains (137c and UVM4). To evaluate the statistical differences in peptide abundance between the two strains, PEAKS software utilises an unpublished algorithm (partially based on the Significance B method used by Cox and Mann (2008)) named PEAKS Q (Lin et al. 2013). The algorithm outputs a fundamental parameter, the significance value (− 10log*P*_value_). Differentially expressed proteins were selected based on the following 3 criteria: (i) fold change (≥ 2), (ii) number of unique peptides detected (≥ 3), and (iii) significance (≥ 20). The significance value was selected as ≥ 20, which is equivalent to a *p*-value ≤ 0.01, to keep a high stringency, as suggested in PEAKS documentation (https://www.bioinfor.com/protein-quantification/). All datasets were exported as.csv files and uploaded onto the UniProt website to obtain a more complete annotation of the proteins. PEAKS identification includes the protein accession number, which matches the UniProt database number. However, redundancies in the database present a significant issue, as the same amino acid sequence can often be found under multiple accession numbers and/or protein names. These redundant annotations were removed manually to simplify the proteomic analysis. Annotations from the PEAKS software identification and the UniProt database annotation were combined to manually categorize the significant proteins based on Gene Ontology (GO): biological process, molecular function and cellular component (Gaudet et al. 2011).

### Glycosylated extracellular protein analysis

The protein and glycoprotein profile of the samples were investigated on a polyacrylamide gel. The amount of total protein was normalized to 100 μg and boiled in 1 × Laemmli sample buffer (Bio-Rad, Hercules, California, USA) containing β-mercaptoethanol (9:1 buffer to β-mercaptoethanol and 3:1 sample to buffer ratio) for 10 min at 95 °C. The denatured proteins were loaded in triplicates on two separate 4–15% Criterion™ TGX™ Precast Gels (Bio-Rad, Hercules, California, USA) and separated according to size by gel electrophoresis in Tris/Glycine/SDS buffer (Bio-Rad, Hercules, California, USA) for 40 min at 300 V and 290 mA alongside a protein ladder (Precision Plus Protein™ Dual Colour Standards, Bio-Rad, Hercules, California, USA) with protein size range of 10–250 kDa. One gel was stained with Coomassie as described by Arndt et al. (2018), while the second gel was used for glyco-staining using the Pierce™ Glycoprotein Staining Kit (Thermo Fisher, Waltham, Massachusetts, USA), following the manufacturer’s protocol. The kit is based on the periodic acid-Schiff (PAS) method with the fuchsine sulphite dye. Positive and negative control proteins (HRP and STI, respectively) were tested previously (data not shown). The Coomassie-stained and fuchsine-stained gels were visualised using the Typhoon FLA 9000 Gel Imager (GE, Chicago, Illinois, USA). The bands of interest were excised from the Coomassie-stained gel for in-gel trypsin digestion as previously described (Raymond et al. 2013). The released proteins were subsequently analysed by tandem mass spectrometry as described above, using the following program: 5–30% MS buffer B (98% acetonitrile + 0.2% formic acid) over 30 min, 30–80% MS buffer B over 3 min, 80% MS buffer B for 2 min, and 80–85% MS buffer B for 3 min. Protein identification was performed as described above.

### Statistical analysis

The statistical analysis of the proteomics results is described above. Statistical analysis of the cell density data was done in GraphPad Prism 5.0 (GraphPad Software, La Jolla, California USA). Kolmogorov–Smirnov and Levene’s tests were first used to confirm normality and homoscedasticity of the data, respectively. A parametric test (two-way ANOVA) followed by a post-hoc Fisher’s least significant difference (LSD) test were used to test the null hypothesis that there was no difference in growth between the 137c and UVM4 strains. The results were considered significant at *p*-value < 0.05. Throughout the paper, values given are mean ± standard error of the mean (SEM) (*n* = 3 biological replicates).

## Results

### Strains 137c and UVM4 show physiological differences

Prior comparing the secretomes of the cell-wall deficient strain UVM4 and its cell-walled parental strain 137c, we analysed and compared their cell morphology, motility, and growth rates to better inform the proteomics results.

**Fig. 1:**
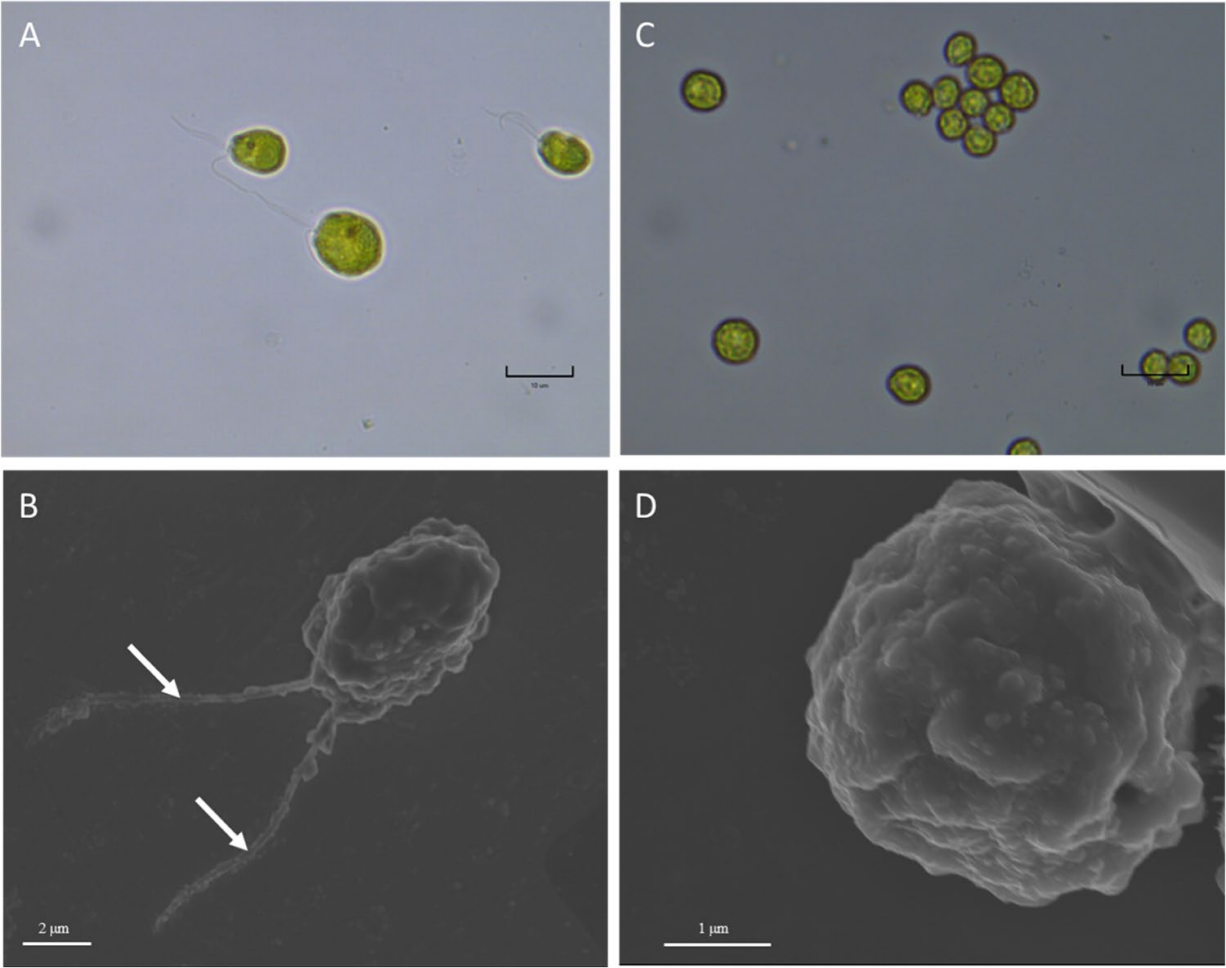
Comparative imaging of *C. reinhardtii* wild type strain (137c; **A** and **B**) and UV-mutated strain (UVM4; **C** and **D**), showing significant difference in size and presence/absence of flagella. Optical image of *C. reinhardtii* wild type strain (137c; **A**) and UV-mutated strain (UVM4; **C**) displaying difference in size, scale bar: 10 μm. SEM micrograph of *C. reinhardtii* wild type strain (137c; **B**) and UV-mutated strain (UVM4; **D**) indicating the presence of two flagella (white arrow) in strain 137c, while absent in strain UVM4. Scale bars: 2 μm (**B**); 1 μm (**D**)

Notable morphological differences were detected between strains 137c and strain UVM4 following microscopic analyses. Strain UVM4 cells were approximately half the diameter of 137c cells, with an average size of 5.8 μm and 10 μm respectively (Fig. 1, Supplementary Fig. S2). The absence of a cell wall and flagella was observed in strain UVM4 (Fig. 1, Supplementary Fig. S2), which are traits consistent with direct descent from strain CC-4350.

**Fig. 2:**
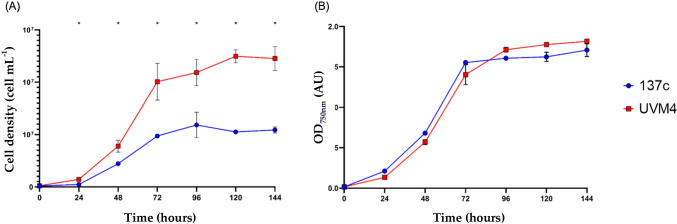
Comparison of 137c and UVM4 strains growth. (**A**) Cell density was monitored by flow cytometry. (**B**) Optical density was measured by spectrophotometry. Samples were collected at 72 h for secretome analysis. Data are mean ± SEM (*n* = 3). ^*^Significant differences between the two strains (Fisher’s LSD, *p* < 0.05)

Considering the differences in size between strain 137c and strain UVM4, cell density (cells mL^−1^) and optical density (at 750 nm) were simultaneously measured to assess differences in growth (Fig. 2).

Although the cell density values for strain 137c and strain UVM4 cells were significantly different, the optical density values were similar due cell size differences (Fig. 2), indicating a similar level of light absorption by cells in culture. We harvested both UVM4 and 137c in late-exponential phase (72 h) for secretome analysis. During the following day (96 h), the two strains showed a slight, although not significant, difference in growth with strain 137c likely entering stationary phase 24 h before strain UVM4 (Fig. 2B).

### Strain UVM4 shows higher abundance of cell wall proteins in the extracellular space

**Fig. 3:**
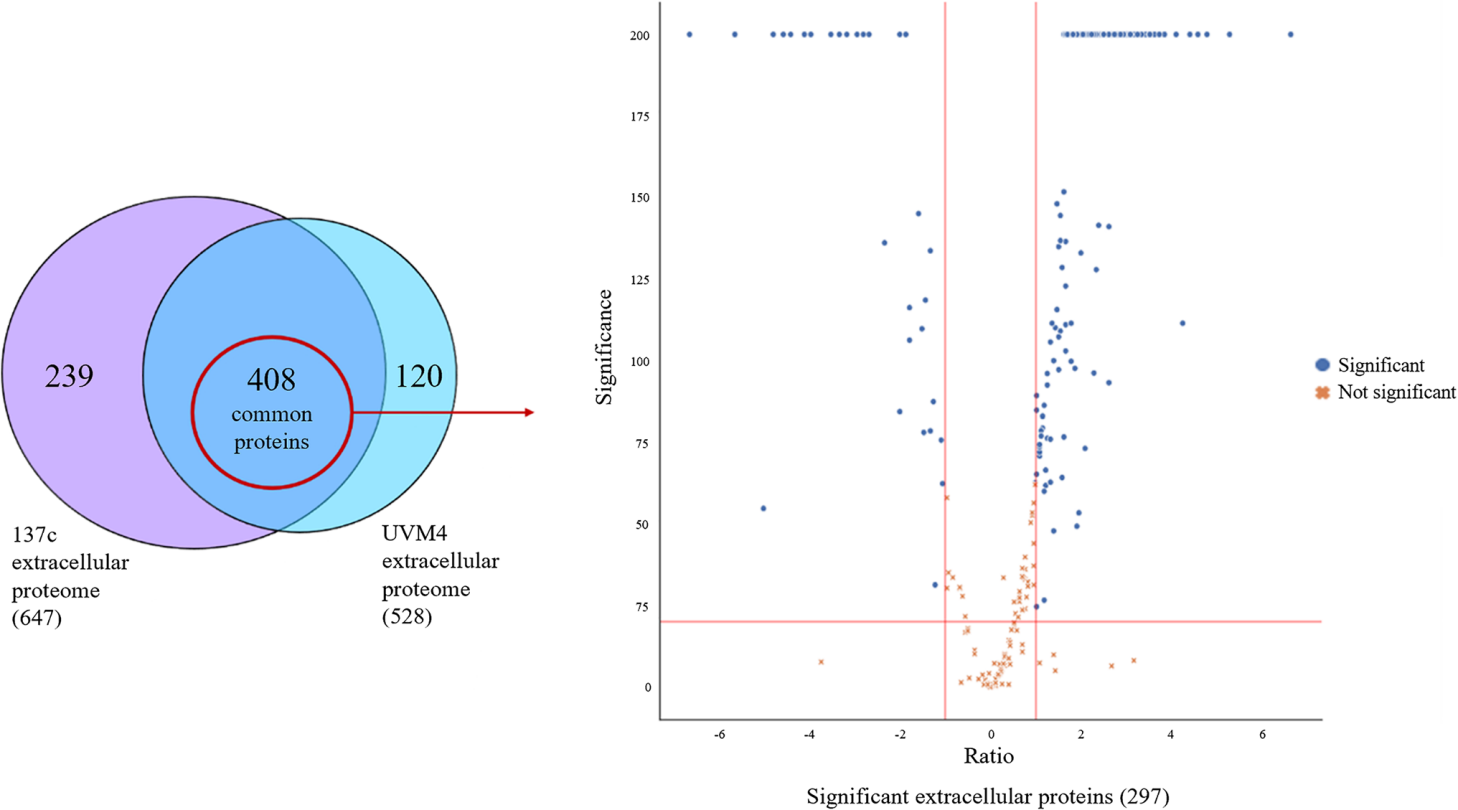
Venn diagram (**A**) of the extracellular proteome comparison between strains 137c and UVM4, and volcano plot (**B**) of significant extracellular common proteins. (**A**) The comparison between the extracellular proteomes of strain 137c (647 total identified proteins) and strain UVM4 (528 total proteins) revealed 408 extracellular proteins present in both secretomes. Amongst these 408 common proteins, only 297 were found to be significantly differentially produced in strain UVM4 compared to strain 137c (represented by blue dots on the volcano plots). (**B**) Differentially produced proteins were selected based on significance value ≥ 20 (equivalent to *p*-value ≤ 0.01) and fold change of − 1 ≥ log_2_ ≥ 1, *n* = 3

The secretomes of strains UVM4 and 137c were harvested at 72 h (OD_750nm_ ≃ 1.5 AU) for proteomic analysis. The proteins detected in all three biological replicates were identified (Supplementary Table S1–S18) and the complete secretome profiles of strains 137c and UVM4 were compared, obtaining common proteins (detected and identified in both strains) and unique proteins (detected and identified only in one strain). A total of 408 extracellular proteins common to both strains were detected (Fig. 3). These proteins were analysed using label-free quantification to obtain their relative abundance in strain UVM4 using strain 137c proteins as a control (Fig. 3). Of these, only the significant proteins (significance value ≥ 20 and fold change − 1 ≥ log_2_ ≥ 1, parameters generated by the PEAKS Q algorithm (Lin et al. 2013)) were investigated further (297 proteins total) (Supplementary Table S19–S20). For the unique proteins, it was not possible to obtain relative abundance using label-free quantification; however, these unique proteomic profiles were examined to validate and support the results obtained by the label-free quantification of the common proteins.

**Fig. 4:**
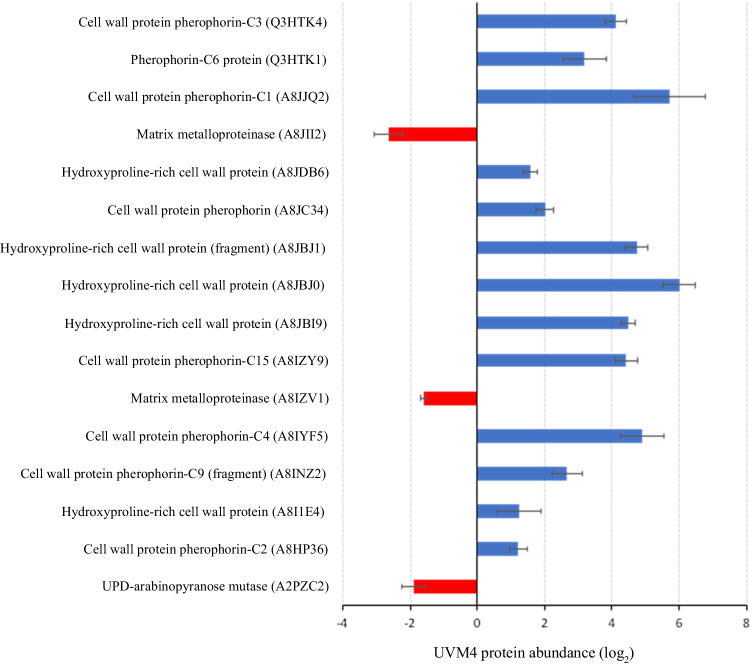
Relative abundance of cell wall proteins in UVM4 using protein abundance in 137c as reference. The relative abundance is based on the average of the triplicates. The proteins showing higher abundance in UVM4 than in 137c are in blue, while the less abundant proteins are in red (data are mean ± SD, *n* = 3 biological replicates). The Uniprot accession numbers of each protein are given in brackets

The proteins were manually annotated based on the Gene Ontology project categories: biological process, molecular function and cellular component (Gaudet et al. 2011) and organised into subgroups based on predicted functional annotation. Amongst the different groups identified (Supplementary Fig. S3), we chose to focus on cell wall proteins (Fig. 4) based on the hypothesis that unassembled cell wall proteins would occur in higher abundance in the secretome of the cell wall–deficient strain UVM4.

In congruence with the literature, many of the differentially produced cell wall proteins were found to be more abundant in strain UVM4 extracellular space. Of these, half were 16–64 (i.e. 2^4^–2^6^) times more abundant in strain UVM4 secretome than the same proteins produced in strain 137c, representing a substantial increase in cell wall proteins being secreted into strain UVM4 growth media. All extracellular cell wall proteins showing differential abundance in strain UVM4 can be grouped in 3 different families: cell wall–specific enzymes (matrix metalloproteinases and carbohydrate mutase), hydroxyproline-rich glycoproteins (HPRGs), and pherophorins (a subclass of HPRGs) (Fig. 4). Interestingly, these groups of proteins show a distinct pattern: all the cell wall–specific enzymes were less abundant in the extracellular space for strain UVM4, while all HPRGs and the pherophorins showed higher abundance.

Considering the overabundance of pherophorins in the extracellular space of strain UVM4 (Fig. 4), we hypothesised that the aggregates identified by Zhang and Robinson (1990) that hinder RP purification consist of these pherophorins. To further understand how these proteins behave in the secretome, we pursued a gel-based strategy to determine whether pherophorins are the HPRG subclass responsible for the protein aggregates.

### Extracellular glycoproteins in strain UVM4 form high molecular aggregates absent in strain 137c

**Fig. 5:**
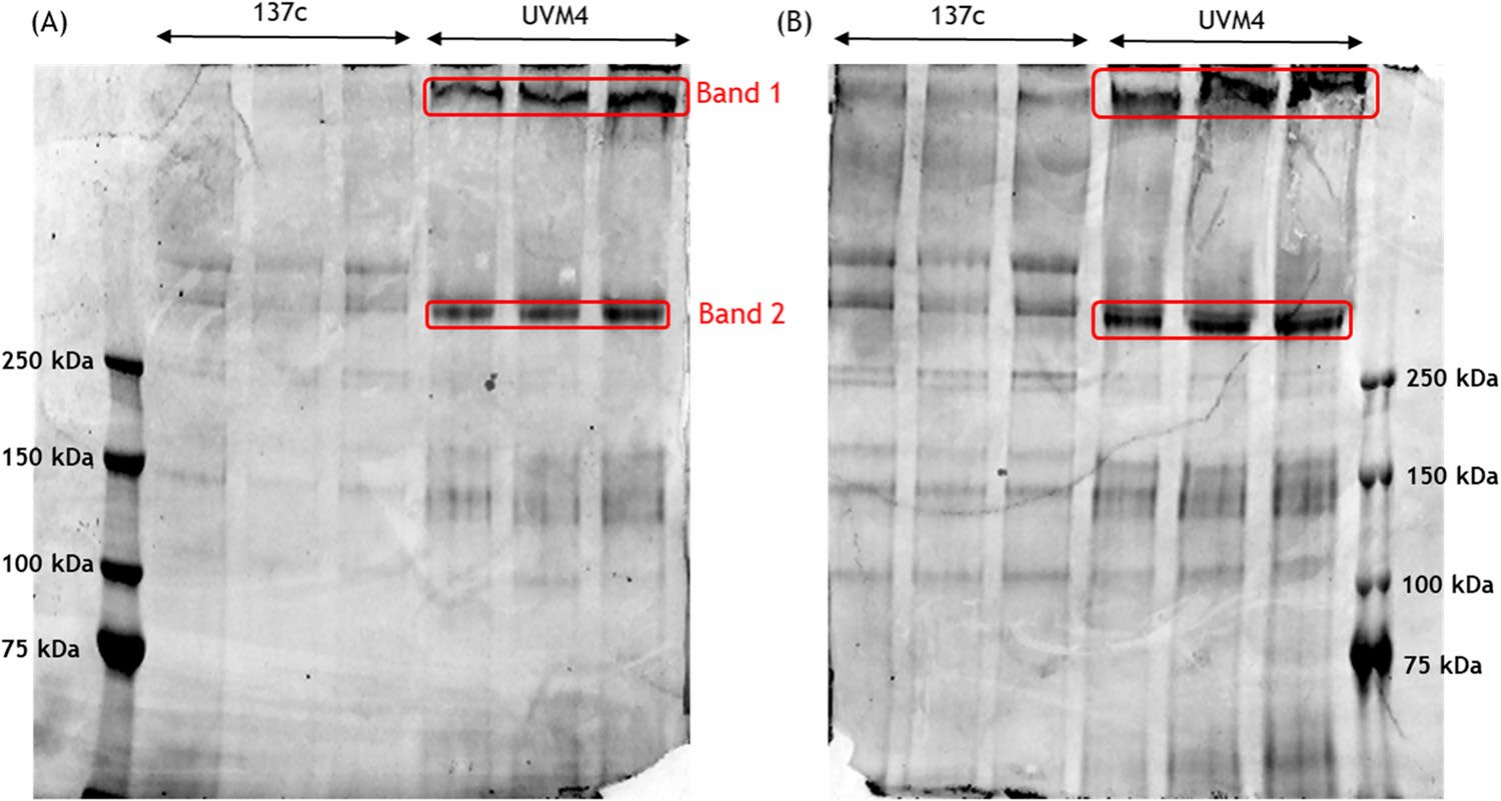
SDS-PAGE gels of strains 137c and UVM4 secretomes stained with (**A**) Coomassie and (**B**) glycoprotein staining. Sample loaded: ~ 100 μg total protein (*n* = 3, biological replicates). Intense bands of high molecular weight proteins in both Coomassie and glyco-stained gels (red squares) suggest overabundance of glycosylated extracellular proteins in strain UVM4 relative to strain 137c. Given that proteins with molecular weight lower than 75 kDa did not show any detectable differences, the gel was run longer to improve the separation of the high molecular weight proteins

Given that cell wall proteins (including pherophorins) are commonly known to be heavily glycosylated, to possibly identify them and investigate their presence/absence, molecular weight, and relative abundance (intensity) in the extracellular spaces of strains 137c and UVM4, two protein gels were performed: (i) a Coomassie-stained gel to detect total proteins in the extracellular spaces of the two strains (Fig. 5A), and (ii) a glycoprotein-stained gel to detect only the glycosylated proteins present in the extracellular spaces of the two strains (Fig. 5B).

The two strains displayed different protein and glycoprotein profiles in the extracellular space. The differences in intensity of the bands between Coomassie staining and glycoprotein staining were expected, considering that the glycostaining is more sensitive and will show bands not visible on the Coomassie stained gel.

Interestingly, almost all the bands visible on the Coomassie stained gel were glycosylated, and the glycoprotein profiles between the two strains showed extensive differences. The major alterations in pattern and intensity of proteins and glycoproteins between the two strains were noticeable for proteins with molecular weight higher than 250 kDa. In particular, two intense bands > 250 kDa (in triplicates) were specifically found in strain UVM4 (red squares, Fig. 5). Given that these glycoproteins showed molecular weight > 250 kDa and higher abundance in the secretome of strain UVM4, we suspected these bands to correspond to the aggregates described by Baier et al. (2018). Therefore, these two intense bands (Band 1 and Band 2, Fig. 5) were excised from the gel and analysed singularly using mass spectrometry.

### High molecular weight bands present in the extracellular space of strain UVM4 are pherophorin aggregates

The identification of the proteins in Bands 1 and 2 (Fig. 5) was performed using the software PEAKS (manually validating the results) and summarized in Tables 1 and 2. All the proteins identified are cell wall proteins, with a majority being pherophorins. Both Band 1 and Band 2 showed multiple protein identifications, and the proteins found in Band 2 are also present in Band 1. In addition, a deeper analysis of these results in comparison with the extracellular proteomic results shown in Fig. 4, showed that three pherophorins identified in the gel bands (A8HP36, A8JJQ2, and Q3HTK4) (Tables 1 and 2) were also showing higher abundance in the label-free quantification of the secretome of strain UVM4 (Fig. 4). Specifically, proteins A8JJQ2 and Q3HTK4 were respectively 52- and 17-fold more abundant in strain UVM4. The other two proteins identified in the two bands (A8HP37 and Q6PLP6) were also detected by label-free quantification; however, their abundance in the two strains was not significantly different.

**Table 1:** Identified proteins from gel band 1, specifically found in strain UVM4

Accession number	Protein	#Peptides	#UniqueP	Avg. mass (kDa)
A8HP37	Cell wall protein pherophorin-C13	66	18	52.9
A8HP36	Cell wall protein pherophorin-C2	55	7	52.6
A8JJQ2	Cell wall protein pherophorin-C1	26	15	50.1
Q6PLP6	Cell wall protein GP2 (fragment)	20	20	120.3
Q3HTK4	Cell wall protein pherophorin-C3	14	14	47.1

**#Peptides**: The number of peptide sequences that are present in a protein group

**#UniqueP**: The number of peptide sequences that are unique to a protein group

**Table 2:** Identified proteins gel band 2, specifically found in strain UVM4

Accession number	Protein	#Peptides	#UniqueP	Avg. mass (kDa)
Q6PLP6	Cell wall protein GP2 (fragment)	86	86	120.3
A8HP36	Cell wall protein pherophorin-C2	13	3	52.6

**#Peptides**: The number of peptide sequences that are present in a protein group

**#UniqueP**: The number of peptide sequences that are unique to a protein group

Both bands analysed are of a molecular weight greater than 250 kDa, while all the identified proteins have molecular weights equal or lower than 120 kDa. However, we have high confidence in the identity of the proteins found in each band, given the high number of identified peptides and multiple hits for the same peptide (often unique peptides were identified multiple times). Therefore, the data supports the hypothesis that the high molecular weight bands are largely comprised of pherophorin protein aggregates. The absence of a smear between Band 1 and 2 shows that the aggregates are of different but well-defined sizes.

## Discussion

This study provides the first proteomic analysis of the secretome of *C. reinhardtii* strain UVM4, with a focus on cell wall proteins secreted into the extracellular space. Furthermore, we provide the first evidence that pherophorins are the specific class of HPRGs involved in the formation of extracellular protein aggregates, which are most likely responsible for impaired RP purification due to their unique carbohydrate-binding properties.

Proteomic analysis revealed a lower abundance of cell wall–specific enzymes and a higher abundance of HPRGs in the extracellular space of the cell wall–deficient strain UVM4.

The cell wall–specific enzymes showing lower abundance in strain UVM4 included two metalloproteinases (A8IZV1 and A8JII2) that target and hydrolyse proline- and hydroxyproline-rich cell wall proteins. Given the role of these metalloproteinases, it is possible that their lower abundance in the secretome of strain UVM4 is related to accumulation of HPRGs and pherophorins in the extracellular space. The UDP-arabinopyranose mutase (UAM; UniProt accession number: A2PZC2) also had lower abundance in strain UVM4 and has been experimentally characterised in *C. reinhardtii* (Kotani et al. 2013). UDP-arabinopyranose mutase is an isomerase involved in the provision of UDP-L-arabinofuranose for the glycosylation of HPRGs found in the *C. reinhardtii* cell wall. The lower abundance of this protein in strain UVM4 may be related to the absence of an assembled cell wall to anchor the generated polysaccharides. A topic of further investigation would be to determine if the arabinosylation status of extracellular HPRGs are affected by this observed reduction in UAM.

As shown in Fig. 4, two classes of cell wall proteins reported higher abundance in the extracellular space of strain UVM4: HPRGs and pherophorins. HPRGs are the major components of *C. reinhardtii* cell wall (Ferris et al. 2001). They present a characteristic repetitive amino acidic backbone, are rich in hydroxyproline residues, and are heavily glycosylated (Ferris et al. 2001; Sommer-Knudsen et al. 1998). This class of proteins can be divided in multiple subclasses, based on their structural motifs and cellular functions (Sommer-Knudsen et al. 1998; Showalter 1993). Due to their repetitive protein domains, HPRGs are predominantly involved in cell wall meshwork and structural roles (Sommer-Knudsen et al. 1998). However, some HPRG subclasses, such as solanaceous lectins and pherophorins, are able to perform more complex functions (Sommer-Knudsen et al. 1998; Showalter 1993; Hallmann 2006; Godl et al. 1995). In fact, solanaceous lectins are carbohydrate-binding proteins involved in multiple roles, including sugar transport, cell–cell interaction, defence mechanisms, and wound healing (Sommer-Knudsen et al. 1998; Showalter 1993). Pherophorins are fundamental cell wall proteins involved in stress response and sexual induction and development (Hallmann 2006; von der Heyde and Hallmann 2020; Godl et al. 1995). These proteins have been extensively characterised in *Volvox*, another green alga. However, given that *Volvox* and *C. reinhardtii* belong to the same order (Chlorophyceae), it is predictable that the function of pherophorins will be identical in *C. reinhardtii*. The ability of these classes of cell wall proteins to perform such vital and complex roles in chlorophytes lies in their advanced protein structure (Godl et al. 1995; Hallmann 2006; Showalter 1993; Sommer-Knudsen et al. 1998). In fact, both solanaceous lectins and pherophorins present heavily glycosylated hydroxyproline and serine repeats in a rod-like shape with two globular domains at both ends (Hallmann 2006). It has been experimentally proven that these globular domains, called globular A and B domains, are responsible for the carbohydrate-binding function in solanaceous lectins (Van Damme et al. 2004), and it has been postulated that they may have the same role in pherophorins (Hallmann 2006). Unfortunately, this particular protein shape and the carbohydrate-binding activity of the two globular domains may be responsible for the experimentally reported formation of pherophorins and lectins aggregates (Ender et al. 2002; Ueno et al. 1991; Hallmann 2006; von der Heyde and Hallmann 2020). In fact, it has been hypothesised that their unique dumbbell-like shape, with two carbohydrate-binding domains separated by long heavily glycosylated hydroxyproline-rich spacers, coupled with the specific sugar-to-protein bonding mechanism of their globular domains, could lead to inter-molecular cross-linking, forming the protein aggregates (Ender et al. 2002; Ueno et al. 1991; Hallmann 2006; von der Heyde and Hallmann 2020).

The presence of cell wall HPRG aggregates in the extracellular space of cell wall–deficient strains of *C. reinhardtii* has been previously reported (Zhang and Robinson 1990). However, our approach implicates pherophorins as the specific class of HPRGs and identifies specific proteins (Table 1 and 2) involved in the formation of extracellular aggregates.

Although our data reveals the composition of these aggregates, more information is needed regarding the interaction between the pherophorin clusters and the secreted RPs. Unveiling the nature of chemical-physical interaction of pherophorins and RPs will be important information to help avoid the entrapment of the bioproduct and consequent RP purification difficulties.

The novel insights presented in this study can be used to inform future strategies to mediate the formation of extracellular protein aggregates in *C. reinhardtii* strain UVM4. These include possible chemical approaches to detach the multiple linked pherophorins, or genetic engineering approaches to reduce pherophorin abundance using knock-down or knock-out methodologies. These diverse approaches may improve downstream purification processes, possibly resulting in higher yields of RPs and facilitating the industrial deployment of *C. reinhardtii* as a biofactory.

## Supplementary Information

Below is the link to the electronic supplementary material.

Supplementary file1 (PDF 468 KB)

Supplementary file2 (XLSX 9947 KB)

## Data Availability

All data generated and analysed during this study are included in this published article as supporting material.
