# Supplementary material for: Unassembled cell wall proteins form aggregates in the extracellular space of Chlamydomonas reinhardtii strain UVM4
Source: Appl Microbiol Biotechnol. 2022 May 23;106(11):4145–56. doi: 10.1007/s00253-022-11960-9 (PMC9200674; doi:10.1007/s00253-022-11960-9)
Supplement: Supplementary file 1 — Supplementary file1 (PDF 468 KB) [file 253_2022_11960_MOESM1_ESM.pdf]

Applied Microbiology and Biotechnology

Unassembled cell wall proteins form aggregates in the extracellular space of *Chlamydomonas reinhardtii* strain UVM4

Lorenzo Barolo[1*] (0000-0002-9640-0740), Audrey S. Commault[1] (0000-0002-6730-0089), Raffaela M. Abbriano[1] (0000-0002-5754-5461), Matthew P. Padula[2] (0000-0002-8283-0643), Mikael Kim[1] (0000-0001-7059-9432), Unnikrishnan Kuzhiumparambil[1] (0000-0003-0582-6779), Peter J. Ralph[1] (0000-0002-3103-7346) and Mathieu Pernice[1] (0000-0002-3431-2104)

*lorenzobarolo@gmail.com Climate Change Cluster, University of Technology Sydney, Sydney, NSW, Australia

[1] Climate Change Cluster, University of Technology Sydney, 15 Broadway, Ultimo 2007, Sydney, NSW, Australia

[2] School of Life Sciences and Proteomics Core Facility, Faculty of Science, University of Technology Sydney, 15 Broadway, Ultimo 2007, Sydney, NSW, Australia

SUPPLEMENTARY FIGURES

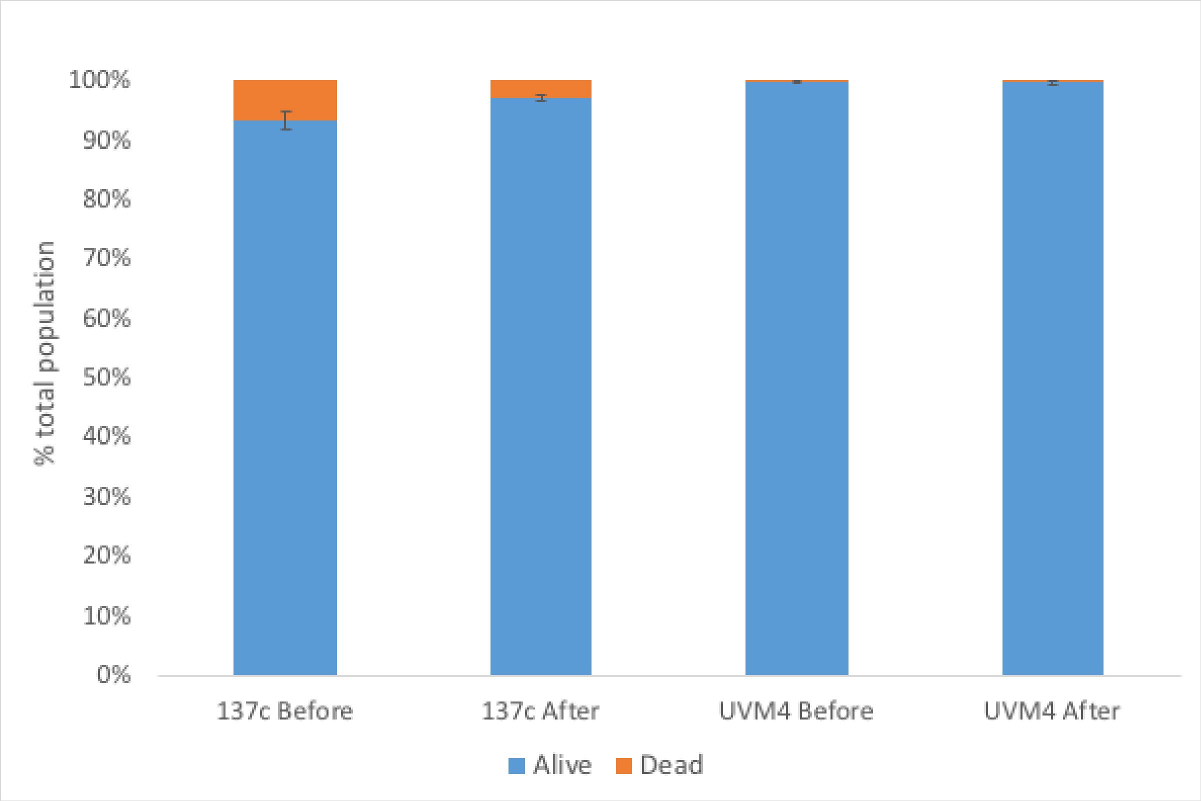

Supplementary Fig. S1: Percentage of living and dead cells for strains 137c and UVM4 before and after filtration. An aliquot of cells (1 mL) before and after the filtration was stained using the LIVE/DEAD® Fixable Violet Dead Cell Stain (ThermoFisher Scientific, Waltham, Massachusetts, USA) and analysed by flow cytometry. Using live and dead controls, it was possible to measure the number of dead cells in the sample before and after the filtration, and subsequently calculate the difference (%) of dead cells after the treatment. The results show no increase in dead cells after the filtration. The proportion of dead cells in 137c after filtration became lower, potentially related to dead cells breaking open during the filtration process due to weaker cell walls. However, that represented only ~3.5% (6.5 – 3 %) of the total population, which is unlikely to have significantly contaminated the secretome.

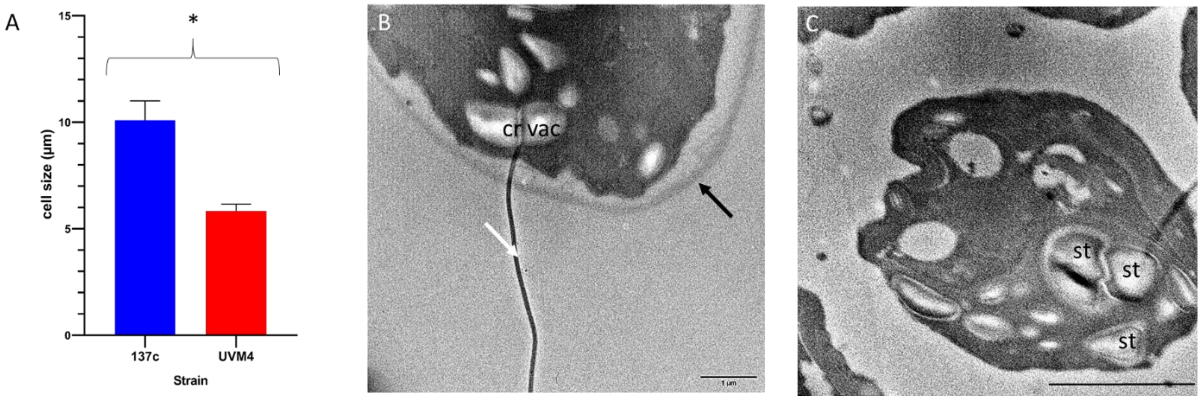

Supplementary Fig. S2: Cell size comparison of strains 137c and UVM4 (A) based on microscopic images (*significant differences between the two strains, $p < 0.05$, n = 30). Comparative TEM micrograph of *C. reinhardtii* wild type strain (137c; B) and UV mutated strain (UVM4; C) indicating the presence of cell wall (black arrow) and flagellum (white arrow) in 137c strain, while absent in UVM4. Scale bar: 1 µm (B); 2 µm (C). st, starch; cr vac, contractile vacuoles.

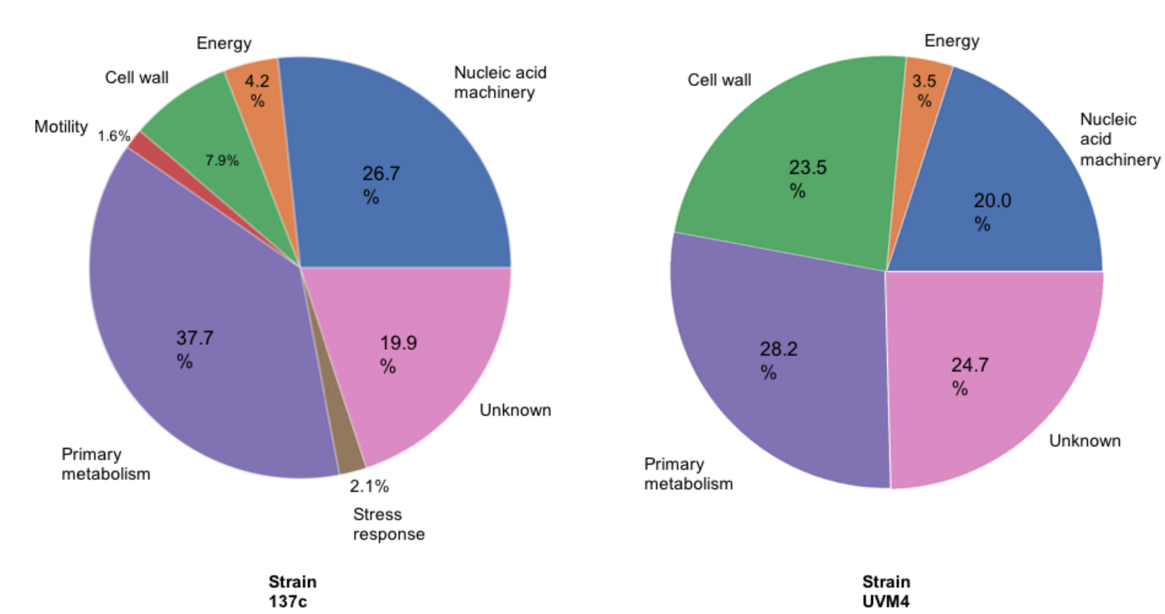

Supplementary Fig. S3: Proteins present in the secretomes of strains 137c and UVM4 categorised based on Gene Ontology (GO): biological process, molecular function and cellular component (Gaudet et al. 2011). Cell wall proteins represent almost a quarter (23.5%) of strain UVM4 total secretome, while only 7.9% in strain 137c secretome.
